# Effects of Gamma Irradiation on Bacterial Microflora Associated with Human Amniotic Membrane

**DOI:** 10.1155/2013/586561

**Published:** 2013-08-26

**Authors:** Fahmida Binte Atique, Kazi Tahsin Ahmed, S. M. Asaduzzaman, Kazi Nadim Hasan

**Affiliations:** ^1^Department of Biochemistry and Microbiology, School of Life Sciences, North South University, Bangladesh; ^2^Tissue Banking and Biomaterial Research Unit, Atomic Energy Research Establishment, Savar, Bangladesh

## Abstract

Human amniotic membrane is considered a promising allograft material for the treatment of ocular surface reconstruction, burns, and other skin defects. In order to avoid the transmission of any diseases, grafts should be perfectly sterile. Twenty-five amniotic sacs were collected to determine the microbiological quality of human amniotic membrane, to analyze the radiation sensitivity pattern of the microorganism, and to detect the radiation decimal reduction dose (D_10_) values. All the samples were found to be contaminated, and the bioburden was ranged from 3.4 × 10^2^ to 1.2 × 10^5^ cfu/g. Initially, a total fifty bacterial isolates were characterized according to their cultural, morphological, and biochemical characteristics and then tested for the radiation sensitivity in an incremental series of radiation doses from 1 to 10 KGy. The results depict gradual decline in bioburden with incline of radiation doses. *Staphylococcus* spp. were the most frequently isolated bacterial contaminant in tissue samples (44%). The D_10_ values of the bacterial isolates were ranged from 0.6 to 1.27 KGy. *Streptococcus* spp. were found to be the highest radioresistant strain with the radiation sterilization dose (RSD) of 11.4 KGy for a bioburden level of 1000. To compare the differences, D_10_ values were also calculated by graphical evaluations of the data with two of the representative isolates of each bacterial species which showed no significant variations. Findings of this study indicate that lower radiation dose is quite satisfactory for the sterilization of amniotic membrane grafts. Therefore, these findings would be helpful to predict the efficacy of radiation doses for the processing of amniotic membrane for various purposes.

## 1. Introduction

Transplantation with a wider array of tissues of human origin had been practiced for many years to achieve lifesaving and enhancing consequences. The role of amniotic membrane in transplantation is well established because of their unique characteristics and properties [1]. Amnion is the innermost layer of the placenta that lines the amniotic cavity, shows a thickness of 20–50 *μ*m, and is much stronger than the chorion [2]. In Bangladesh, amniotic membrane allografts have been used over the past 20 years for various clinical disorders ranging from—management of burn as a biological dressing, as a substrate to replace the damaged ocular tissues and skin injuries—acid violence, leprotic ulcer, bedsore, traumatic open wound, and so forth [3]. The beneficial effects of amniotic membrane graft transplantations in ophthalmology for corneal diseases (chemical injury, persistent epithelial defects, and corneal ulceration) and conjunctival diseases (the Stevens-Johnson syndrome, conjunctival cicatrisation, and conjunctivochalasis) have also been reported. The use amnion was evidenced for extremity ulcerations results from long-term diabetes and surgical wounds [4]. Amniotic membrane grafts gain importance because of their anti-inflammatory, antiangiogenic, antifibrotic, and antiscarring properties [5]. Because of the pliability and elasticity, the amnion membrane grafts could be very easily applied onto the wound areas and their application is not associated with immunological problems [6]. Transplanted graft reduces pain, stimulates healing, and shows better scar appearances. Furthermore, the amniotic membrane could be prepared rather inexpensively compared to the other biological dressings and possesses most of the characteristics of an ideal skin substitute [7]. As the membrane comes in contact with open wound, its microbiological quality is the most important consideration [8]. In order to avoid the transmission of diseases, the membrane needs be perfectly sterile and should bear high sterility assurance level (SAL). Sterilization of tissue allograft is generally performed by using chemicals, heat, UV, gamma radiation, and E-beam. Among them, gamma radiation from ^60^Co sources had been considered to be the most desirable method for tissue sterilization [9]. When gamma radiation is applied to the amniotic membrane grafts for sterilization, the process is thought to be superior than chemical sterilization. As the bioburden associated with amniotic membrane may vary from specimen to specimen, it is important to assess general radio sensitivity of the microorganisms and to detect highly resistant microbes [10]. In Bangladesh, information about the type and magnitude of microbial contamination related with amniotic membrane grafts is limited. Therefore the present study was addressed to determine the microbiological quality of human amniotic membrane, to analyze the radiation sensitivity pattern of the microorganism, and to detect the radiation decimal reduction dose (*D*_10_) values.

## 2. Materials and Methods

### 2.1. Tissue Procurement

Twenty-five amniotic membrane sacs were collected from the healthy mothers after normal vaginal deliveries from different hospitals of the Dhaka city. All the donors were prescreened for the presence of transmissible diseases (e.g., HIV, HBV, and VDRL). Amnion samples were collected (glossy, translucent, and thinner membrane) from the hospitals under aseptic condition in a plastic container containing normal sterile saline (0.9% Nacl) and preserved temporarily in a freezer below −20°C. During collection each container was labeled with donor ID and hospital registration number. The containers were then placed in a cool box and transported to the tissue banking laboratory as early as possible. The membranes were then washed separately with sterile normal saline several times using orbital shaker to remove blood.

### 2.2. Isolation and Characterization of Bacterial Isolates

Initially, 1.8 gm to 6.43 gm of tissues were cut at different parts of the samples and taken in 100 mL sterile conical flask containing 20 mL sterile physiological saline. Using the orbital shaker, the beaker containing the sample was gently shaken for at least 15 minutes. 10 mL of suspension was taken by sterile pipette into a test tube from the beaker and serially diluted up to 10^−5^. If discrete colonies were not detected in 10^−5^ dilution, further dilutions were prepared and the tests were then repeated. Platting of the samples were performed using five different agar media—nutrient agar, MacConkey agar, eosin methylene blue agar, thioglycoliate agar, and mannitol salt agar base in an aseptic condition using biohazard class-II laminar airflow cabinet. For the better and authentic bacterial count, both spread and pour plate methods were followed with 0.1 mL and 0.3 mL of cell suspension, respectively. All the plates were incubated at 37°C for 24 hours. Cultural characteristics of the bacterial isolates were studied by gram staining using freshly prepared reagents to identify whether the isolates were gram (+)ve or gram (−)ve. The shape, size, form, texture, and pigment production of isolated colonies were observed. Different biochemical analyses including, oxidase, urease and catalase test, indole, TSI, and MRVP test were carried out according to the standard methods of Cheesbrough [11] to identify the isolates.

### 2.3. Radiation Sensitivity Pattern of the Isolates

After isolation and identification, a total of forty-one bacterial isolates of amnion were screened for comparative radiation resistance from 1 to 10 KGy of radiation doses. Bacterial colonies were counted at two stages, before radiation and after radiation. For the untreated population, cells were incubated for 2 to 3 hours at 37°C; platting was performed with appropriate dilutions and again incubated for 24 hours at 37°C, and viable cells were estimated. For the irradiated population, platting was done after the exposure of different radiation doses.

### 2.4. Assay for *D*_10_ Value and Determination of Radiation Sterilization Dose (RSD)

To determine the survival fraction (S) for each radiation dose, the number of viable cells after radiation was divided with the initial viable cell number. For all the strains, survival curves relating to log *S* were obtained with irradiation dose. Finally, the *D*_10_ values or the doses which can reduce the microbial population by 90% were calculated by using the equation *D*_10_ value = *D*/(log No − log N), where *D* is the radiation dose, No is the untreated bioburden, and N is the irradiated bioburden. Graphically *D*_10_ values were calculated by using Excel's linear regression analysis based on the linearity of the survivor curves in which *D*_10_ values were taken as the negative reciprocal of the slope of the regression line [12]. To achieve the sterility assurance level (SAL) of 10^−6^, radiation sterilization dose (RSD) for maximum bioburden of 1000 was determined by the equation RSD = *D*_10_ (Log bioburden − Log SAL) KGy [13].

## 3. Results

All the samples were contaminated, and the initial bioburden was ranged from 3.4 × 10^2^ to 1.2 × 10^5^ cfu/g. Among the bacterial contaminants,* Staphylococcu*s spp. were found to be the highest abundant organism (22 in 50, 44%) while *Pseudomonas* spp. were the lowest (5 in 50, 10%) (Table 1). Preliminarily, a total 50 bacterial isolates were characterized based on their morphological, cultural, and biochemical tests. According to the gram staining, a majority (87.5%) of the microbial contaminants observed on the samples were gram positive in which 75% were cocci shaped. Only 12.5% of the bioburden on the amnion were gram negative rods. No gram negative cocci were found. According to the biochemical tests, all the bacterial isolates were oxidase and citrate negative. Three of the isolates were indole positive, and only one isolate was urease positive cocci.

A total forty-one bacterial isolates were exposed from 1 KGy to 10 KGy gamma radiations from 60 Co gamma sources. Before radiation, the highest count was observed in *Streptococcus *sp. as 3.8 × 10^7^ cfu/mL and the lowest count in *Pseudomonas* sp. as 2.4 × 10^6^ cfu/mL. Among the 41 bacterial isolates, 40 (97.5%) isolates were capable of surviving at 5 KGy, 26 (63.4%) isolates survived up to 6 KGy, 18 (43.9%) isolates survived up to 7 KGy, and only 7 (17.1%) isolates were able to survive at 8 KGy. Even 2 (4.8%) isolates were found to be survive at 9 KGy (Table 2). Gamma irradiation doses equal or greater than 9 KGy were sufficient for the total elimination of the growth and multiplication of bacterial isolates.

The radiation decimal doses (*D*_10_) values for all the bacterial isolates were determined (Table 3). Radiation sterilization dose (RSD) for the bioburden level of 1000 was also calculated. Plotting the logarithm of survival fraction (log_10_ *S*) versus radiation dose (KGy), survival curves for different bacteria were obtained. In this study, relatively higher *D*_10_ values were recorded for the gram positive isolates. The *D*_10_ values for the microbial isolates ranged from 0.6 to 1.27 KGy.

**Figure 1:**
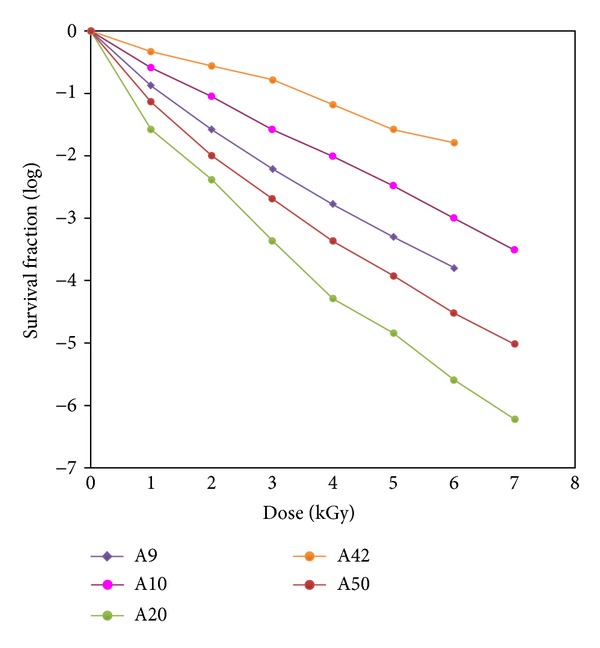
Survival curves of *Bacillus* spp.

**Figure 2:**
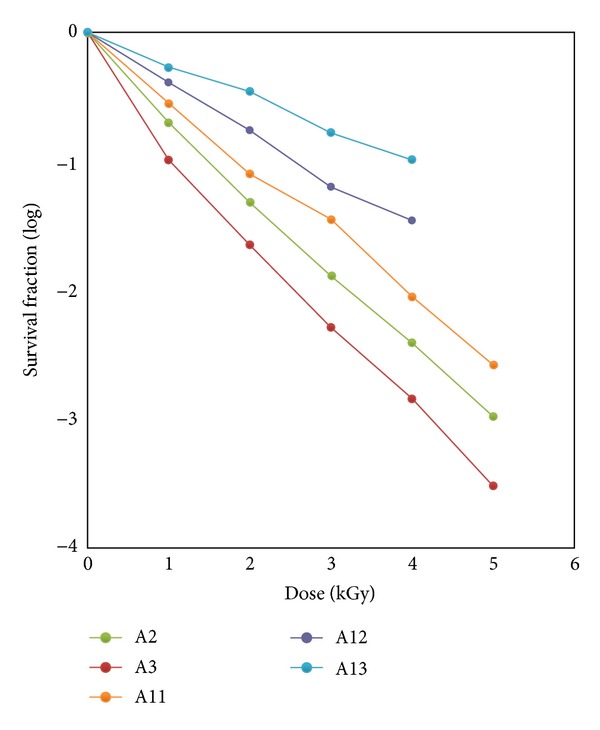
Survival curves of *Pseudomonas* spp.

Different types of microorganisms have different dose response curves, and these differences imply that different microorganisms possess different innate sensitivities to radiation [8]. In our study, a total five isolates of *Bacillus* spp. were tested for their resistance to gamma irradiation. The *D*_10_ values of *Bacillus* spp. was observed between 0.83 KGy and 0.99 KGy (Figure 1). On the other hand, relatively lower *D*_10_ values were recorded for gram negative *Pseudomonas* spp. in the range of 0.60 KGy to 0.74 KGy (Figure 2).

**Figure 3:**
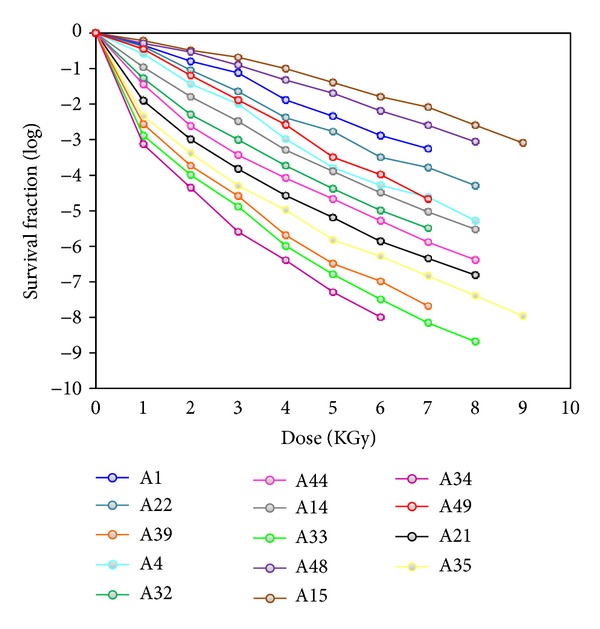
Survival curves of *Streptococcus *spp.

**Figure 4:**
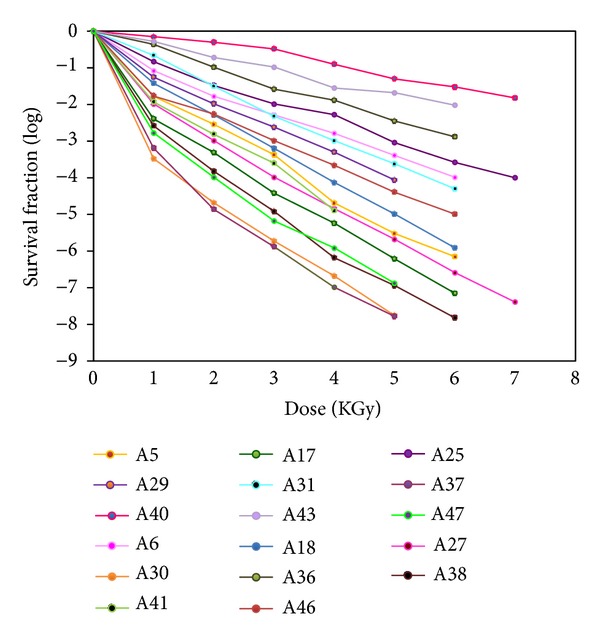
Survival curves of *Staphylococcus* spp.

S*treptococcus *spp. showed the highest *D*_10_ value of 1.06 KGy to 1.27 KGy (Figure 3) in amniotic membrane. Even thereof few isolates of the *Streptococcus *spp. were able to survive at 9 KGy of gamma radiation. The *Staphylococcus* spp. tend to dominate the microbial population in amnion. In this investigation, *D*_10_ value of *Staphylococcus spp. *ranged from 0.83 to 0.97 KGy (Figure 4). Though, Log_10_ *S* value of the isolates at each data point was very close, so overlapping lines were obtained in the survival curve (Figures 3 and 4).

Radiation death of the bacterial isolates was also obtained by the linear regression equations. Linear functions were most significant at the 95% confidence level. Though there were a few fluctuations in *D*values, the overall showed no significant differences.

## 4. Discussion

Tissue bank deals with the human connective tissues for clinical use with the guaranteed quality from the moment of retrieval up to the use as allograft. Though the storage procedure is well documented in tissue banks, the appearances of infection due the bacterial contamination cannot be excluded. As a result of prolonged hospitalization, organ failure or even death can occur. In most cases, infection occurs after graft implant. Despite thorough donor screening, microorganisms could be introduced into the grafts from various sources during tissue procurement, processing, handling, or storage or at the time of surgery. Even pregnant women with preterm labor and intact amniotic membrane could be able to carry microorganisms in their amniotic fluid [14]. The most common microbial isolates from the amniotic cavity from women with preterm labor and intact membranes were found to be *Ureaplasma urealyticum*, *Fusobacterium* spp., and *Mycoplasma hominis*. [15]. Other microorganisms that were found in the amniotic fluid include *Staphylococcus aureus*, *Streptococcus agalactiae, Peptostreptococcus *spp., *Gardnerella vaginalis, Streptococcus viridans,* and *Bacteroides* spp. Approximately one-half of the infections associated with human tissue transplants were found to be due to the bacterial agents, and of that, 90% were aerobic organisms [16]. Along with environmental exposure, underlying diseases and the host defense mechanism can also contribute to the graft contamination in ratio between 2 and 5% [17].

In our study, all the amniotic membrane samples were collected from seronegative (HIV, HBV, and VDRL) donors and were procured under aseptic condition. In spite of following good tissue banking practices, donor tissues were not sterile. Most of the samples were contaminated with *Staphylococcus* spp. and *Streptococcus *spp. Many authors [18, 19] have already summarized the staphylococcal contamination in amnion grafts. According to Tomford et al., [20] 5%–65% graft contamination occurs due to the bacterial strain, and 36% to 38% is caused by coagulase-negative staphylococci, especially *Staphylococcus epidermidis *[21]. Engel et al. [22] had reported coagulase negative staphylococci as major contaminant in amnion allografts. Aghayan et al. [23]also reported staphylococci as their most prevalent organisms and 72.53% of their amnion grafts were contaminated with these organisms.

In order to prevent contamination in allograft strategies like asepsis, proper use of disinfectants, sterilization procedure, or the perioperative administration of systemic antibiotics needs to be taken [24]. Gamma radiation had been the most commonly employed method for the sterilization of tissue allografts because of the several advantageous factors. According to International Atomic Energy Agency [25], a radiation dose of 25 KGy is defined as the reference dose for the sterilization of the tissue grafts, but to keep intact the biomechanical and other properties of tissues, some tissue banks prefer lower radiation dose without compromising SAL 10^−6^. Gamma radiation dose of more than 25 KGy can impair the mechanical properties of grafts, destroy the osteoinductive capacity of tissue, and can reduce tissue graft incorporation. Use of lower radiation sterilization dose will lessen the effects on physical properties of amnion grafts. Baker et al. [26] have found that sterility (10^−6^ SAL) of tissue allograft could be achieved using at least 9.2 KGy, and in another study conducted by Singh et al. [8] showed that 17.6 KGy gamma radiation was substantiated as radiation sterilization dose (RSD) for amnion allografts.

 In this study, bacterial isolates were tested for their survival in radiation doses from 1 KGy to 10 KGy, and relatively higher gamma radiation doses were required for the gram positive bacteria. Among them, the growth of *Streptococcus* spp. was observed even up to 9 KGy, but at 10 KGy all the isolates were killed. On the contrary, all the gram negative isolates were found to be killed after 5 KGy. The findings of this study were found to be in concordance with the other studies where gram negative microbes were more radiation sensitive than gram positive [27]. It might occur due to the differences in lipid content between the cell wall of gram positive and gram negative bacteria [13]. Evaluations of the bioburden showed that most of the samples were contaminated and the initial bacterial load was high; however after radiation treatment, the bacterial load was somewhat decreased. The behavior of the microbial population is highly relevant to the different doses of ionizing radiation [28]. Though a radiation dose of 25 KGy is recommended for the sterilization of tissue, the present study observed that lower radiation doses were quite satisfactory to achieve the desired sterility assurance level.

## 5. Conclusion

Transmission of disease or infection is always a serious concern in allograft transplantation. However, aseptic technique practices in tissue bank can reduce but could not totally eliminate the microbial load. Right choice of radiation sterilization dose would be helpful to limit the infectious diseases and to obtain sterile allografts. This study demonstrated that though a radiation dose of 25 KGy is sufficient for the sterilization of tissue allograft, a lower radiation dose might be acceptable to keep the allograft sterile. Further study might be suggested for the determination of lower RSD using radio resistant reference strain.

## Figures and Tables

**Table 1:** Frequency distribution of microorganisms.

Isolate	Frequency	Percentage
*Staphylococcus *spp.	22	44%
*Streptococcus* spp.	16	32%
*Bacillus *spp.	07	14%
*Pseudomonas *spp.	05	10%

Total	50	100%

**Table 2:** Radiation response of bacterial isolates.

Organisms	Total number of isolates	Survive up to radiation dose (KGy)						
		4	5	6	7	8	9	10
*Staphylococcus *spp.	17	17	16	7	3	Nil	Nil	Nil
*Streptococcus* spp.	14	14	14	14	12	7	2	Nil
*Pseudomonas *spp.	5	5	3	Nil	Nil	Nil	Nil	Nil
*Bacillus *spp.	5	5	5	5	3	Nil	Nil	Nil

Total	41	41	40	26	18	7	2	Nil
% Survival		100%	97.5%	63.4%	43.9%	17.1%	4.8%	Nil

**Table 3:** Ranges of the *D*_10_ values and RSDs of the bacterial isolates.

Organisms	RSD (KGy), bioburden 1000	Sublethal doses (KGy)	*D*_10_ values (KGy)
*Staphylococcus* spp.	3.2–7.5	4–7	0.83–0.97
*Streptococcus* spp.	4.9–11.4	7–9	1.06–1.27
*Pseudomonas* spp.	1.2–3.3	4-5	0.60–0.74
*Bacillus* spp.	4.5–9.4	6-7	0.83–0.99
